# Age Related Response of Neonatal Rat Retinal Ganglion Cells to Reduced TrkB Signaling *in vitro* and *in vivo*

**DOI:** 10.3389/fcell.2021.671087

**Published:** 2021-06-04

**Authors:** Jamie Beros, Jennifer Rodger, Alan R Harvey

**Affiliations:** ^1^School of Biological Sciences, The University of Western Australia, Crawley, WA, Australia; ^2^Perron Institute for Neurological and Translational Science, Nedlands, WA, Australia; ^3^School of Human Sciences, The University of Western Australia, Crawley, WA, Australia

**Keywords:** developmental cell death, neurogenesis, retinal ganglion cells, superior colliculus, brain-derived neurotrophic factor, TrkB, retinotectal

## Abstract

During development of retinofugal pathways there is naturally occurring cell death of at least 50% of retinal ganglion cells (RGCs). In rats, RGC death occurs over a protracted pre- and early postnatal period, the timing linked to the onset of axonal ingrowth into central visual targets. Gene expression studies suggest that developing RGCs switch from local to target-derived neurotrophic support during this innervation phase. Here we investigated, *in vitro* and *in vivo*, how RGC birthdate affects the timing of the transition from intra-retinal to target-derived neurotrophin dependence. RGCs were pre-labeled with 5-Bromo-2′-Deoxyuridine (BrdU) at embryonic (E) day 15 or 18. For *in vitro* studies, RGCs were purified from postnatal day 1 (P1) rat pups and cultured with or without: (i) brain derived neurotrophic factor (BDNF), (ii) blocking antibodies to BDNF and neurotrophin 4/5 (NT-4/5), or (iii) a tropomyosin receptor kinase B fusion protein (TrkB-Fc). RGC viability was quantified 24 and 48 h after plating. By 48 h, the survival of purified βIII-tubulin immunopositive E15 but not E18 RGCs was dependent on addition of BDNF to the culture medium. For E18 RGCs, in the absence of exogenous BDNF, addition of blocking antibodies or TrkB-Fc reduced RGC viability at both 24 and 48 h by 25–40%. While this decrease was not significant due to high variance, importantly, each blocking method also consistently reduced complex process expression in surviving RGCs. *In vivo*, survival of BrdU and Brn3a co-labeled E15 or E18 RGCs was quantified in rats 24 h after P1 or P5 injection into the eye or contralateral superior colliculus (SC) of BDNF and NT-4/5 antibodies, or serum vehicle. The density of E15 RGCs 24 h after P1 or P5 injection of blocking antibodies was reduced after SC but not intraretinal injection. Antibody injections into either site had little obvious impact on viability of the substantially smaller population of E18 RGCs. In summary, most early postnatal RGC death in the rat involves the elimination of early-born RGCs with their survival primarily dependent upon the availability of target derived BDNF during this time. In contrast, late-born RGC survival may be influenced by additional factors, suggesting an association between RGC birthdate and developmental death mechanisms.

## Introduction

During development of the peripheral nervous system and at least some regions of the central nervous system (CNS), populations of cells experience naturally occurring cell death (Oppenheim, 1991). RGCs are one such CNS population; in the rat at least 50% of RGCs produced in the embryo die during pre- and early postnatal development, much of it occurring within the first 5 postnatal days (Dreher et al., 1983; Perry et al., 1983; Crespo et al., 1985). It is generally held that the phenomenon of naturally occurring cell death is associated with the regulation of cell number and the refinement of structural morphology and connectivity during development. The mechanisms that regulate such death remain unclear, but misguidance of axons and competition for limited target-derived trophic support are likely to be contributing factors (Purves, 1988).

Rat RGCs are first generated in the retina at about E13, the last cells differentiating at approximately E21 (Bunt et al., 1983; Crespo et al., 1985; Reese and Colello, 1992; Rapaport et al., 2004). Upon differentiation, RGCs project axons via the optic nerve to a variety of central targets (Sefton et al., 2015). The great majority of RGCs terminate in the contralateral SC, with about 40% also sending branches into the dorsal visual thalamus (Linden and Perry, 1983; Simon and O’Leary, 1992; Ahmed et al., 1996; McLaughlin et al., 2003). RGCs that are produced early (E13-E16) have axons already present in the SC by the time of birth (P0), whereas axons from their late-born counterparts (E18-E19) do not grow into the SC until about P4/P5 (Dallimore et al., 2002; Dallimore et al., 2010).

Developing neurons rely on neurotrophic support for their survival and maturation (Huang and Reichardt, 2001). Perhaps the most well studied neurotrophin in RGC development is BDNF, whose neuroprotective effects on RGCs have been documented *in vivo* and *in vitro* (Johnson et al., 1986; Ma et al., 1998; Spalding et al., 2004; Moses et al., 2015). BDNF is synthesized and can be released as pro-BDNF to act on the p75 receptor, or the pro domain can be cleaved to produce mature BDNF that acts on the high affinity tropomyosin receptor kinase B (TrkB) receptor to bring about neuroprotective effects (Nagappan and Lu, 2005; Yang et al., 2009). BDNF and TrkB mRNA and proteins are detected in early embryonic development in RGCs, the levels of expression changing throughout development (Ernfors et al., 1992; Jelsma et al., 1993; Koide et al., 1995; Perez and Caminos, 1995; Vecino et al., 2002; Moses et al., 2015). However, neurotrophins are also produced in the SC and can be retrogradely transported to the retina via RGC axons (Ma et al., 1998; Frost et al., 2001; Spalding et al., 2002, 2004). Thus, while there are local intra-retinal sources of BDNF (De Araujo and Linden, 1993; Cellerino and Kohler, 1997; Marler et al., 2010) and these can also be rapidly transported anterogradely (Spalding et al., 2002), it is generally thought that RGC survival is eventually dependent on competition for limited quantities of target-derived BDNF, a general mechanism that is theorized to match neuronal populations with the size of their targets (Purves, 1988; Davies, 1994, 1996).

The requirement for neurotrophins during RGC development is well documented. *In vivo*, once RGC axons have innervated the SC, removal of the SC target or reducing local neurotrophins such as BDNF and NT-4/5 that act on the TrkB receptor, results in rapid and extensive RGC death (Harvey and Robertson, 1992; Cui and Harvey, 1995; Spalding et al., 2004). Similarly, when culturing developing RGCs, the absence or reduced availability of exogenous neurotrophins such as BDNF and ciliary neurotrophic factor (CNTF) reduces RGC viability (Johnson et al., 1986; Meyer-Franke et al., 1995; Moses et al., 2015). However, neurotrophin dependence seems to be exhibited only by RGCs whose axons have already innervated their target, and the timing of RGC loss reflects the timing of innervation of central targets (Dallimore et al., 2002, 2010). There is evidence that RGCs whose axons have not yet reached the SC can survive independently of BDNF, presumably relying on local, intraretinal expression of neurotrophins. Moses et al. (2015) used BrdU, a nucleoside analog that incorporates into cellular DNA during synthesis, to prelabel early (E15) or late-born (E18) RGCs and performed qPCR analysis on these RGCs at different postnatal ages to assess changes in mRNA expression of genes implicated in survival and development. Late-born RGCs at P1 with axons *en route* to their target have high expression of BDNF and genes associated with downstream signaling of TrkB that are implicated in axon outgrowth and survival. At P5, patterns of gene expression in this cohort changed to resemble those of their early-born counterparts, with axons in the target since P0. Additionally, Moses et al. (2015) linked prior innervation of the SC with exogenous neurotrophin dependence *in vitro* by culturing birthdated RGCs at P1. These results showed that late born RGCs with their axons not yet in the SC at P1 were able to survive independently of exogenous BDNF 24 and 48 h after plating, complementing the identified changes in gene expression. Conversely, earlier born RGCs with axons in the SC at the time of cell culture experienced a reduction in cell viability when exogenous BDNF was not added to the culture medium. This suggests that the reliance on target derived neurotrophins is not uniformly experienced by all RGCs during development and differs according to RGC age and timing of axonal innervation of their targets.

In the present *in vitro* and *in vivo* studies, the aim was to further elucidate neurotrophic dependence in BrdU labeled early (E15) or late-born (E18) RGCs by examining the effects of inhibition of TrkB signaling. *In vitro*, the survival of purified, birthdated E15 or E18 RGCs was quantified 24 or 48 h after plating in the presence or absence of BDNF and NT-4/5 blocking antibodies (Cohen et al., 1994; Ghosh et al., 1994; Spalding et al., 2004; Rosenthal and Lin, 2014). We also tested the effect of application of a TrkB-Fc chimera, a TrkB ligand scavenger previously demonstrated to selectively bind neurotrophins with affinity for the TrkB receptor and block its biological activity (Shelton et al., 1995; Vaynman et al., 2004; Schildt et al., 2013; Mariga et al., 2015). *In vivo*, to measure the relative impact of TrkB blockade within the retina or centrally, the viability of identified Brn3a positive (+) E15 or E18 RGCs was assessed 24 h after injection of BDNF and NT-4/5 blocking antibodies into either the retina or SC of rats, at either P1 or P5 (Spalding et al., 2004). In sum, we observed a difference in the response of RGCs to the anatomical origin of trophic support, a response that differed according to RGC birthdate and developmental timeline. In the rat, most early postnatal RGC death involves the loss of early-born rather than late-born RGCs. The viability of early-born RGCs during this time appears dependent primarily upon the availability of target derived BDNF, whereas late-born RGC survival may also be influenced by additional supporting factors.

## Materials and Methods

### Animals

Time mated female Wistar rats were sourced from the Animal Resources Centre (ARC, Perth) at either E14 or E15 days of gestation (day after overnight mating = E0) and housed at the Pre-Clinical Facility at the University of Western Australia (UWA). Rats were maintained under a 12 h light/dark cycle at 21°C and 50% humidity with food and water *ad libitum.* At E15 or E18, rats were anesthetized with isoflurane (4% induction and 2% maintenance) and administered an intraperitoneal injection of BrdU (50mg/kg of maternal body weight) three times during the day (9 a.m., 1 p.m., 5 p.m.) to ensure a sustained period of bioavailability (Dallimore et al., 2010). All procedures were approved by the UWA Animal Ethics Committee.

### Dissociation and Purification of RGCs for Culture

Each cell culture run was created from the pups from one pregnant dam. Parturition occurred on E22/22.5 (day of birth = P0). At P1, pups were euthanized with an overdose of sodium pentobarbital (Lethabarb), eyes removed and retinas dissected and pooled in Dulbecco’s phosphate buffered saline (dPBS). Retinas were dissociated using the MACS neural dissociation kit (Miltenyi Biotec), as per manufacturer’s instructions. RGCs were isolated using the MACS RGC Isolation kit (Miltenyi Biotec) following standard depletion and selection protocols and seeded at an average of 25,000 cells into each well of an 8 well chambered culture slide (Corning^TM^ Falcon^TM^). RGCs were resuspended in neurobasal media containing B27 supplement, triiodothyronine, transferrin, progesterone, sodium selenite, n-acetyl cysteine, bovine serum albumin, L-glutamine, sodium pyruvate, insulin penicillin/streptomyosin and forskolin. RGCs were separated into samples which were resuspended either with or without exogenous BDNF (50 ng/ml; Peprotech) and further subdivided into groups that were resuspended with or without TrkB-Fc (TrkB-Fc^+^, 500 ng/ml; BioSensis PE–1235–20) or with or without a blocking cocktail (Block^+^, 500 ng/ml) containing 1:1 of antibody to rh BDNF:lgG (BioSensis S-015-500) and antibody to rh NT4:lgG (Biosensis S-059-500).

TrkB-Fc mimics the endogenous TrkB receptor and has previously been reported to reduce TrkB receptor activation in cultured cells through binding to and capturing molecules that are ligands of TrkB (Shelton et al., 1995; Coull et al., 2005). In contrast, the antibodies specifically bind to their targeted neurotrophin, depleting their availability and therefore transiently inhibiting TrkB receptor activation. Utilization of antibodies in this way has been verified to reduce downstream signaling of TrkB (Ghosh et al., 1994; Lepack et al., 2016), reduce immunoreactivity within neurons for these neurotrophins (Zhou and Rush, 1996), reduce TrkB phosphorylation (Jiang et al., 2003) and prevent BDNF induced changes in physiology and behavior (Balkowiec and Katz, 2000; Graham et al., 2007; Peters et al., 2010; Rosas-Vidal et al., 2014; Rosenthal and Lin, 2014; Lepack et al., 2015). Furthermore, this same antibody cocktail has been shown to significantly increase RGC pyknosis when injected into the SC or eye of neonatal rat pups (Spalding et al., 2004).

After resuspension, RGCs were seeded onto poly-d-lysine and mouse type-1 laminin coated 8-well culture slides (average of about 25,000 RGCs per well). RGCs were cultured for 24 or 48 h at 37°C, the 48 h condition supplemented with an additional application of TrkB-Fc at 24 h. Each culture run generated enough RGCs for two 8-well chambered slides, one slide each for the 24 and 48 h time points. Each slide contained a combination of all treatment groups (BDNF^–^Block^–^, BDNF^–^Block^+^, BDNF^+^Block^–^, BDNF^+^Block^+^), except for the E18 aged group, where TrkB-Fc replaced the neurotrophin block in some runs (BDNF^–^TrkB-Fc^+^, BDNF^+^TrkB-Fc^+^). E15 cultures were replicated twice and E18 cultures were replicated four times with the neurotrophin block and three times for TrkB-Fc.

### Immunohistochemistry and Quantification of Cell Cultures

At 24 or 48 h, cells were fixed in 4% paraformaldehyde and washed with PBS before labeling with anti-βIII-tubulin antibody (BioLegend, 1:4,000 at 4°C overnight). βIII-tubulin is a class of tubulin proteins comprising the microtubules that form the cytoskeleton of neurons (Breuss et al., 2017) and is as a robust marker to identify cultured RGCs at our assessed ages (Pimentel et al., 2000; Moses et al., 2015). Wells were washed with 0.1 M phosphate buffered saline (PBS) and goat anti-rabbit Alexa Fluor 488 (Sigma, 1:400) was applied for 30 min at room temperature. Cells were then washed with 0.1 M PBS before applying 2 M hydrochloric acid (HCl) for 30 min at 37°C, after which, cells were washed with 0.1 M PBS, then incubated overnight at 4°C with anti-BrdU antibody (Roche, 1:100) in diluent containing 4% NGS, 3% BSA and 0.3% Triton X-100. Cells were then washed with 0.1 M PBS and incubated with donkey anti-mouse Alexa Fluor 555 antibody (Sigma, 1:400) for 2 h at room temperature before washing and mounting in Fluoromount-G (Southern Biotech).

**FIGURE 1:**
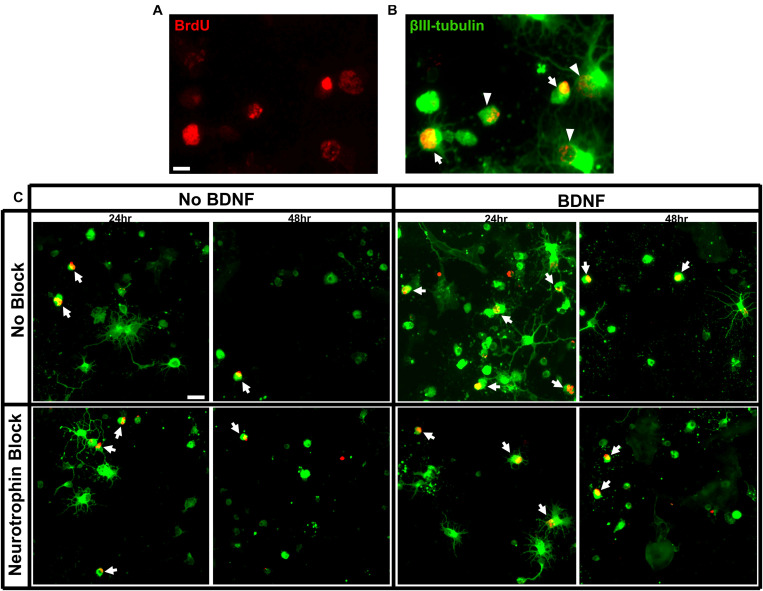
Criteria for RGC quantification and immunolabeling of purified E15 RGC cultures. **(A)** RGCs labeled for BrdU and **(B)** a merged image of RGCs labeled with BrdU and βIII tubulin. Arrows indicate double-labeled RGCs with strong BrdU labeling that would be included in our counts and arrow heads indicated double-labeled RGCs that would be excluded. Scale bar = 10 μm. **(C)** Representative images of RGC cultures at 20x magnification demonstrating the proportion of birthdated E15 RGC to non-birthdated RGCs from all treatment conditions. Arrows indicate quantified BrdU^+^/BIII tubulin^+^ double-labeled RGCs. Note the expression of elaborate βIII tubulin^+^ processes by a proportion of RGCs. Scale bar = 20 μm.

Wells were imaged on a fluorescent microscope at 20x magnification and images captured via a digital camera through NIS elements. Each 70 mm^2^ well was systematically sampled at uniform intervals across the entire well, collecting images with equally distributed distances between. The resulting summed sampled area of each well was 16.18 mm^2^, approximately 23% of each well. The experimenter was blinded to the identity of the well and every cell within the captured sampling area was counted. βIII-tubulin^+^ cells, βIII-tubulin^+^ and BrdU^+^ double-labeled cells (>50% of nuclear area expressing BrdU) were manually counted within each image (Figures 1A,B). The total number of birthdated BrdU^+^ RGCs was expressed as a percentage of the total number of βIII-tubulin^+^ RGCs present in each well and averaged for each treatment group. The proportion of surviving RGCs was expressed as a percentage of the number of initially seeded neurons to provide an indication of overall cell viability. Birthdated RGCs were analyzed as a percentage of the RGCs present at the time of counting to control for variability in RGC numbers between wells and to assess the response of specific aged RGC populations to different experimental conditions compared to the total unlabeled population. A previous study noted that, irrespective of birthdate, the proportion of viable βIII-tubulin^+^ RGCs that expressed multiple, elaborate neurites was greater when BDNF was present in the culture medium (Moses et al., 2015). Here we counted the proportion of all RGCs that possessed three or more βIII-tubulin^+^ processes, at least one of which showed branching and extended at least twice the diameter of the host cell body.

Percentages from each well were averaged across treatment and age groups, with each culture well considered a biological replicate. The final number of wells sampled for each group at 24 and 48 h is presented in Table 1. In sum, 125 wells (64 at 24 h and 61 at 48 h) were analyzed and 23% of each well was sampled; a total of 83,144 RGCs (irrespective of birthdate) was counted across all treatment and age groups. Of these, 2,006 BrdU labeled, βIII-tubulin^+^ E15 RGCs were counted (average of about 63 RGCs counted per well), and 846 double-labeled E18 RGCs were counted (average of about 9 RGCs counted per well).

**TABLE 1:** *In vitro* study: summary of treatment groups and number of culture wells analyzed.

Treatment	E15		E18	
		
	24 h	48 h	24 h	48 h
No BDNF, no Ab block	4	4	8	7
No BDNF, Ab block	4	4	10	10
No BDNF, TrkB-Fc	–	–	6	6
BDNF, no Ab block	4	4	8	6
BDNF, Ab block	4	4	10	10
BDNF, TrkB-Fc	–	–	6	6
Total	16	16	48	45

### Inhibiting TrkB Receptor Activation *in vivo*

At P1 or P5, pups received a 1 μl injection of either normal goat serum or the same blocking cocktail used *in vitro*, containing anti BDNF and anti NT-4/5 (1:1, each at 500 ng/ml). The injection was made into the vitreous of the eye or into the superficial SC using a glass micropipette attached to a 10 μl Hamilton syringe. Each surgery session involved injecting all the pups from one litter, with multiple pups assigned to different treatment groups (for example, some pups received a vehicle injection into the eye at P1 while others received a blocking antibody injection). These treatments were repeated in at least one additional litter so that the treatment groups in the final analysis contained retinas from different litters and surgery days. No eye injection was administered to E15 birthdated pups at P5 as previous studies have shown that RGC axons are present in the SC target at P0 and are dependent on target derived neurotrophins (Bunt et al., 1983; Moses et al., 2015). Prior to surgery, pups were anesthetized via hypothermia before transferring to a plasticine mold mounted on ice to maintain low temperature. For intravitreal injections, the right eyelid was cut to expose the eye and the injection administered close to the ora serrata, avoiding the lens. The eye was cleaned of excess solution and the eyelid left to heal. For SC injections, the skull was exposed along the sagittal midline. A flap was made in the skull above the SC (Spalding et al., 2004; Beros et al., 2018) and injections were targeted to the SGS, the superficial portion of the SC where the majority of RGC axons terminate (Langer and Lund, 1974; Lund et al., 1980). Injections were administered slowly and the pipette remained in the SC for at least 30 s before removal. After injections, the bone flap was replaced and skin sutured. Post-surgery, pups were placed on a warming pad with bedding from the nest until full recovery. Prior to the return of warmed pups to the dam, wounds were cleaned of any fluids.

### Histological Processing of Retinal Sections

Twenty-four hours following P1 or P5 injections, pups were euthanized with an overdose of sodium pentobarbital (Lethabarb, i.p.), transcardially perfused with 4% paraformaldehyde and their eyes and brains collected. Injected eyes, and the eyes contralateral to SC injections, were processed. A dorsal cut was made into the eyes which were additionally post-fixed for 1 h before storage in 0.1 M PBS with 0.1% sodium azide. Prior to sectioning, the cornea and lens were removed and the eye cup (including the retina and sclera) was cryo-protected in 30% sucrose in 0.1 M PBS for 24 h, after which, the solution was graduated with approximately 50% OCT for an additional 24 h. Transverse sections of each eye were cryosectioned at 12 μm from the ventral to dorsal pole and all sections from the entire eye were mounted on Superfrost Plus slides (Lomb Scientific) in serial sections collected in 10 series from the dorsal to ventral retina, taking note to mount each section in relation to its dorsal orientation. One series from each eye was used for immunostaining.

Sections were washed in 0.1 M PBS and membranes were permeabilized in 0.2% Triton-X in 0.1 M PBS for 10 min before blocking with 10% normal donkey serum, 1% bovine serum albumin and 0.2% Triton-X in 0.1M PBS for 1 h. After blocking, sections were incubated in blocking serum containing goat anti Brn3a antibodies (Sigma, 1:200) overnight at 4°C. We used Brn3a as a marker to identify RGCs as the previously used βIII-tubulin antibody labels processes in addition to the nucleus, which can obstruct cell quantification in dense postnatal retinal tissue. Brn3a is a transcription factor localized to the nucleus and is expressed mostly by contralaterally projecting RGCs, with previous studies estimating that Brn3a^+^ cells make up most (approx. 96%), but not all of SC back labeled cells and is expressed at our assessed developmental ages (Quina et al., 2005; Nadal-Nicolás et al., 2009; Voyatzis et al., 2012; Schlamp et al., 2013). The day after primary antibody incubation, sections were washed in 0.1 M PBS before incubation with donkey anti goat Alexa Fluor 488 (1:600) for 2 h at room temperature. Sections were then washed and underwent the same method of anti-BrdU immunolabeling as described for cell cultures, however, after applying donkey anti mouse Alexa Fluor 555, sections were washed in 0.1 M PBS and counterstained with Hoechst (1:1,000) in 0.1 M PBS for 5 min. After washing in 0.1 M PBS, sections were mounted in Fluoromount-G (Southern Biotech).

### Imaging and Quantification of Retinal Sections

To avoid bias associated with over counting in tangential and peripheral sections, images were taken from sections in which the retinas were present as complete arches, not as unbroken circles. Each transverse section contained the entire nasal to temporal axis and a 40x image was taken at a random nasal, central and temporal location for each section sampled, in addition to a low magnification (4x) image of the entire section for measurement and orientation. Every second retinal section was sampled, resulting in an average of 8 sampled sections for eyes injected at P1, and 10 sampled sections for eyes injected at P5.

The experimenter was blinded to the identity of the retinal sections and from the 40x images, a count was made. Only in focus RGCs entirely contained within a section were counted, and only those neurons located in the ganglion cell layer and not in more outer layers or in the inner nerve fiber layer. The likelihood of over counting was minimized by not counting consecutive sections, and by not counting incomplete or fragmentary Brn3a^+^ profiles.

To calculate retinal surface area, the sum of the length of each section at 4x was multiplied by section thickness, sampling rate and series. The surface area of each image at 40x was measured for RGC density calculations which was averaged for each eye. Densities were calculated as Brn3a^+^ RGCs/mm^2^ or BrdU^+^Brn3a^+^ RGCs/mm^2^. *In vivo* data were expressed as a density for consistency with published studies investigating RGC numbers in retinal tissue and for biological relevance. Birthdated RGCs were additionally displayed as a percentage of total RGCs to highlight differences in the survival of specific aged RGCs compared to the total population.

For retinal injections, the eye that received the injection was analyzed and for SC injections, the eye contralateral to the injected SC was analyzed. Injections were conducted on pups from 11 litters that were birthdated at E15 or E18. Each treatment group in the final analysis contained retinas obtained from 2 separate litters, for a total of 63 analyzed retinas as detailed in Table 2.

**TABLE 2:** *In vivo* study: summary of treatment groups and number of retinas analyzed.

Age	Injection location	E15		E18	
			
		Vehicle	Block	Vehicle	Block
P1	Retina	4	6	3	3
	SC	4	6	4	8
P5	Retina	–	–	3	7
	SC	3	3	4	5

### Statistical Analysis

Statistical analyses were conducted using SPSS statistical software package (IBM). For *in vitro* analyses, a 2 × 4 univariate ANOVA was conducted for time (24 and 48 h) and treatment (BDNF^–^ Block^–^, BDNF^–^ Block^+^, BDNF^+^ Block^–^, BDNF^+^ Block^+^) for E15 data. For E18 and data irrespective of RGC birthdate, a 2 × 6 univariate ANOVA was conducted for time (24 and 48 h) and treatment (BDNF^–^ Block^–^, BDNF^–^ Block^+^, BDNF^–^ TrkB-Fc^+^, BDNF^+^ Block^–^, BDNF^+^ Block^+^, BDNF^+^ TrkB-Fc^+^). For *in vivo* analysis, a 2 × 4 univariate ANOVA was conducted for injection group (P1 retina, P1 SC, P5 retina, P5 SC) and treatment (vehicle and blocking cocktail) for E15, E18 and data irrespective of RGC birthdate. Where main or interaction effects were present, Sidak corrected *post hoc* testing was conducted for pairwise comparisons. Significance was determined where α < 0.05. Where appropriate, analysis was conducted on log transformed data to conform to statistical assumptions. For the presented graphs, data are displayed as the mean with error bars representing the standard error of the mean.

## Results

### *In vitro* Experiments

**FIGURE 2:**
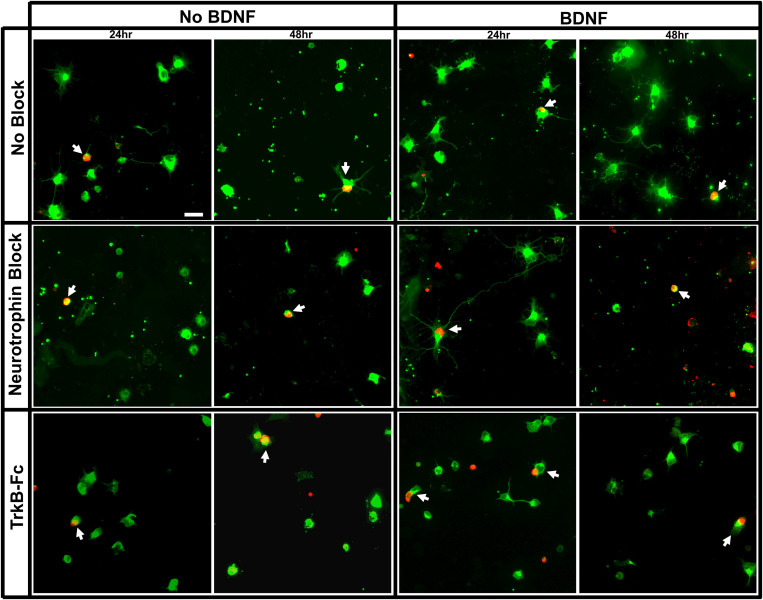
Immunolabeling of purified E18 RGC cultures. Representative images of RGC cultures at 20x magnification demonstrating the proportion of birthdated E18 RGC to non-birthdated RGCs from all treatment conditions, labeled for βIII tubulin (green) and BrdU (red). White arrows indicate BrdU^+^/BIII tubulin^+^ double-labeled RGCs. Scale bar = 20 μm.

All culture wells contained purified βIII-tubulin^+^ RGCs with a distinct subpopulation that was also immunopositive for BrdU. The proportion of surviving RGCs that was double labeled for BrdU and βIII-tubulin was much greater in the E15 compared to E18 injected cohorts (arrows, Figures 1, 2). While there were many nuclei that contained some amount of BrdU incorporation, RGCs were only counted as BrdU^+^ if at least 50% of their nucleus contained robust staining (Figure 1B) (Moses et al., 2015). Cells with low amounts of fragmentary BrdU label were excluded from counts of birthdated RGCs as they may have been daughter cells that had divided sometime after the final *in vivo* BrdU injection (Packard Jr., Menzies and Skalko, 1973; Jose and Soriano, 1989).

**FIGURE 3:**
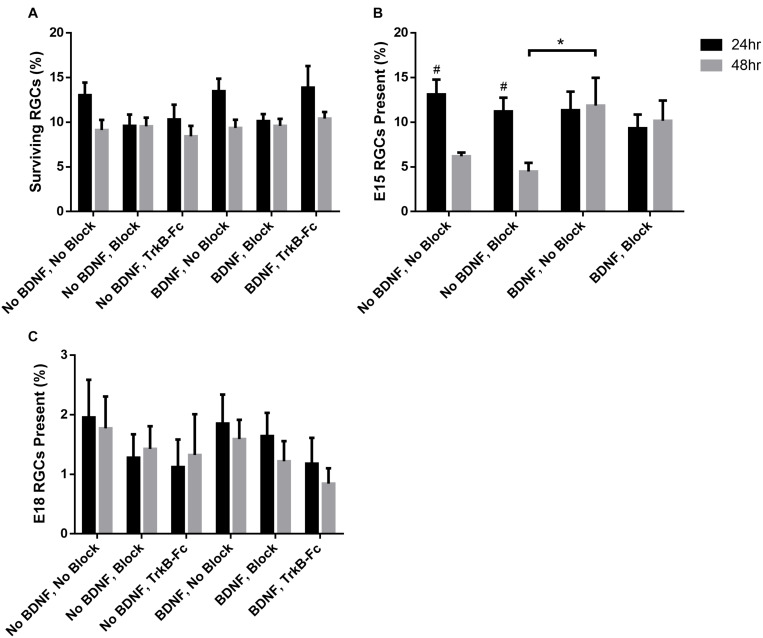
Percentage of surviving RGCs in different treatment groups assessed 24 and 48 h after plating. **(A)** The percentage of the total surviving RGCs from the amount seeded irrespective of birthdate. **(B)** Percentage of surviving E15 and **(C)** E18 birthdated RGCs of those present at 24 and 48 h. * Denotes significant Sidak corrected pairwise comparisons (α < 0.05) between groups and # denotes significant pairwise comparisons between 24 and 48 h within treatment groups.

On average, about 25,000 RGCs were seeded in each of the 8 wells per chamber slide. The proportion of plated RGCs, irrespective of birthdate, that remained viable in each well ranged from 9.60 ± 1.26% to 13.88 ± 2.41% at 24 h, and 8.42 ± 1.18% to 10.41 ± 0.72% at 48 h, post-seeding (Figure 3A). There was thus a small but consistent reduction in overall RGC survival between 24 and 48 h, a decrease that was evident in all treatment groups [*F*(1, 113) = 7.49, *p* < 0.01)].

When assessing the proportion of surviving RGCs that displayed multiple, elongated processes 24 h after seeding (examples shown in Figures 1, 2), addition of recombinant BDNF by itself significantly increased elaboration when compared to control wells [*F*(5, 113) = 4.26, *p* < 0.01, Sidak *p* < 0.05]. Importantly, this BDNF induced elaboration was diminished at 48 h in the presence of the blocking cocktail and TrkB-Fc fusion protein, indicating that our blocking methods worked as intended to reduce TrkB downstream signaling.

### *In vitro*, E15 Labeled RGCs

While the percentage of viable E15 RGCs was not significantly different between all treatment groups at 24 h post-seeding (black bars, Figure 3B), wells that lacked exogenous BDNF displayed a significant reduction in E15 RGC survival at 48 h post-seeding [*F*(3, 24) = 3.04, *p* < 0.05; Sidak *p* < 0.05; # in Figure 3B]. Importantly, addition of BDNF to the culture medium greatly enhanced E15 RGC viability at 48 h, an effect that reached significance when this group was compared to cultures lacking BDNF and containing blocking antibodies (asterisk, Figure 3B). Overall, the application of blocking antibodies slightly reduced E15 RGC survival at both 24 and 48 h in the absence of exogenous BDNF, and also at 48 h when BDNF was present. These results indicate that exogenous BDNF, presumably signaling via the TrkB receptor, is required for the sustained viability of cultured E15 RGCs for at least 48 h after plating.

### *In vitro*, E18 Labeled RGCs

As expected (Reese and Colello, 1992; Rapaport et al., 2004; Moses et al., 2015), the proportion of purified, cultured RGCs that were born on E18, and thus the absolute number of surviving BrdU^+^/βIII-tubulin^+^ E18 RGCs, was considerably less than that measured in the E15 cohort. Across all treatment groups, the proportion of E18 RGCs at 24 h ranged between 1.12 ± 0.46% and 1.95 ± 0.63% and at 48 h ranged between 0.84 ± 0.26% and 1.77 ± 0.54% (Figure 3C). Ninety-three wells were prepared containing RGCs from E18 injected pups—about 2.3 million seeded cells in total. Based on raw counts obtained from analyzing 23% of each culture well, there was an estimated average of 37 BrdU^+^ E18 RGCs per well.

Consistent with earlier data (Moses et al., 2015), and unlike E15 RGCs, sustained E18 RGC viability at 48 h did not require exogenous BDNF. However, the application of blocking antibodies and similarly TrkB-Fc reduced E18 RGC survival approximately 25–40% at 24 and 48 h. These trends are consistent with at least some blockade of BDNF that is generated by the RGCs themselves (Moses et al., 2015). However, despite the large number of cultures generated for this age cohort, the consistently low number of double-labeled RGCs was associated with increased variability between biological replicates, and thus reduced statistical power. As a consequence, for the E18 RGC cohort, we were unable to demonstrate any significant differences between treatment groups [*F*(5, 81) = 0.99, *p* = 0.43], or over time [*F*(1, 81) = 0.13, *p* = 0.72]. Note that in the E18 group, and again unlike the E15 group, addition of exogenous BDNF had no additional beneficial impact on RGC viability at 48 h.

### *In vivo* Experiments

To extend the *in vitro* findings and further understand temporal changes in the dependence of RGCs on target derived factors, we examined *in vivo* the survival of birthdated RGCs within the neonatal rat retina with or without application of BDNF and NT-4/5 blocking antibodies. To do this, the antibody cocktail or control serum were micro-injected either into an eye or superficial SC at P1 or P5 and the effect on identified BrdU^+^ E15 or E18 RGC survival then quantified 24 h later.

### *In vivo* Total RGC Counts (Independent of RGC Birthdate)

**FIGURE 4:**
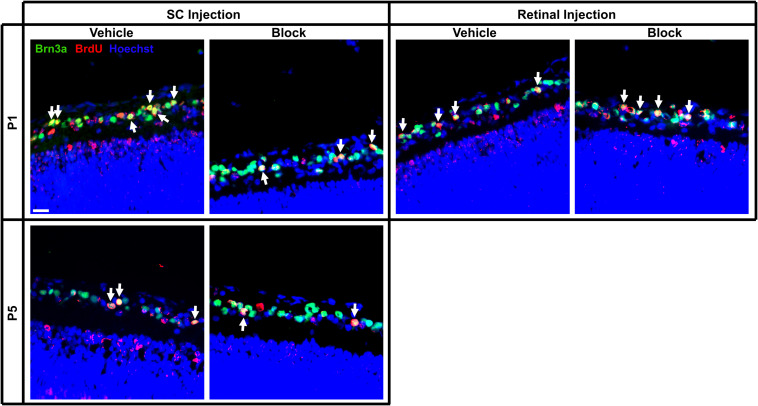
Immunolabeling of E15 RGCs in postnatal rat retinal tissue after P1 or P5 injections of vehicle or blocking antibodies into the retina or SC. Sections were immunolabeled with the nuclear label Hoechst 33342, the RGC marker Brn3a and birthdate identifying BrdU. Images were captured at 40x magnification and channels merged to quantify the amount of BrdU^+^/Brn3a^+^ double-labeled RGCs (arrows). Each image is obtained from the central area of a retinal section, and this section was located at the same approximate location within a series of consecutive sections of an entire eye. Scale bar = 20 μm.

**FIGURE 5:**
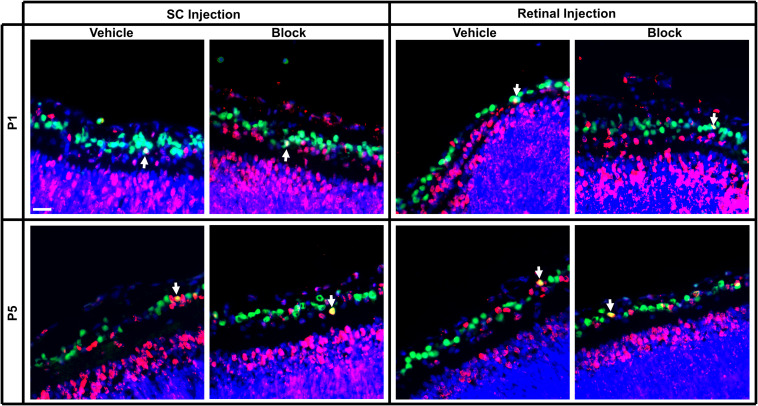
Immunolabeling of E18 RGCs in postnatal rat retinal tissue after P1 or P5 injections of vehicle or blocking antibodies into the retina or SC. Sections were immunolabeled with the same combination of antibodies as Figure 4 and E18 birthdated RGCs were quantified (white arrows). Images were obtained of retinal sections taken from the same approximate location in the series as Figure 4. Note that fewer E18 RGCs were located here in the central retina than in more peripheral retina. Scale bar = 20 μm.

All retinal sections were immunolabeled for Brn3a to identify RGCs (Brn3a^+^), E15 or E18 birthdate (Brdu^+^), and counterstained with Hoechst; RGC numbers were quantified within the ganglion cell layer (Figures 4, 5). Note here that, in accord with earlier studies (McCall et al., 1987), the average retinal area increased with postnatal age, estimated from our sections to change from an average of 14.5 ± 0.6 mm^2^ at P2 to 19.8 ± 1.0 mm^2^ at P6. As expected, the average overall density of RGCs irrespective of birthdate after vehicle injections was significantly reduced with increased age [*F*(3, 55) = 3.03, *p* < 0.05; Sidak < 0.01], from an average of 13,261 ± 562 RGCs/mm^2^ at P2 to 6,132 ± 428 RGCs/mm^2^ at P6. The decrease in density in the overall number of Brn3a^+^ RGCs was greater than might be predicted if it was due only to areal expansion and also reflects ongoing RGC death with increased postnatal age. Importantly, injection of blocking antibodies into the SC at P1 significantly decreased RGC density to 10,889 ± 692 RGCs/mm^2^, a reduction of approximately 20% when compared to controls (Sidak < 0.05). These findings are consistent with earlier studies (Spalding et al., 2004) and reinforce the reliance of RGCs on TrkB signaling and related downstream pathways in the early postnatal period (Moses et al., 2015).

### *In vivo* E15 RGCs

**FIGURE 6:**
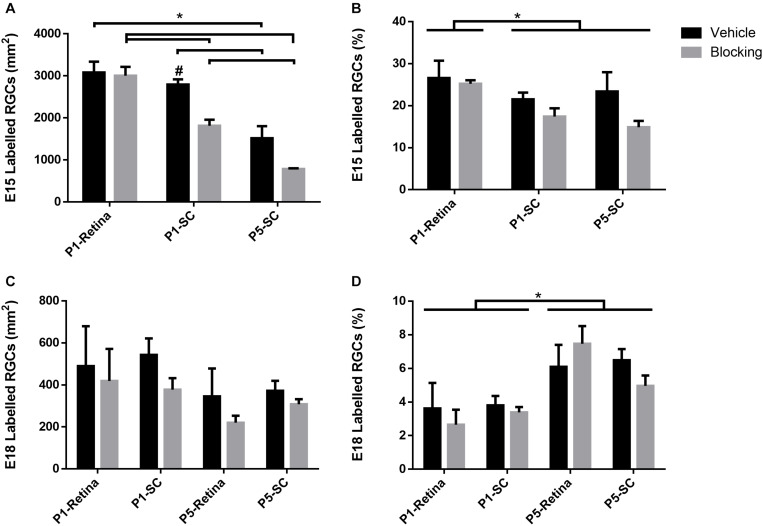
Density and percentage of double labeled (Brdu^+^/Brna^+^) E15 and E18 RGCs in retinal sections after injection of serum (vehicle), or BDNF and NT-4/5 blocking antibodies, at P1 or P5. **(A)** Average density of E15 RGCs and **(B)** average percentage of all RGCs born on E15. **(C)** Average density of E18 RGCs and **(D)** average percentage of all RGCs born on E18. Tailed significance bars denote significant Sidak corrected pairwise comparisons (α < 0.05) of the interaction effect (denoted by *) and bars lacking tails indicate significance of the injection group main effect. ^#^Denotes significant Sidak corrected pairwise comparisons between vehicle and block within injection groups.

When assessing E15 RGCs in retinal sections, a reduction in density was observed with increased developmental age [*F*(2, 20) = 6.02, *p* < 0.01; Sidak < 0.01; Figure 6A]. Furthermore, injection of blocking antibodies into the SC at P1 and P5 significantly reduced E15 RGC densities compared to serum controls (Sidak, *p* < 0.01; # in Figure 6A), whereas blocking antibodies injected into the eye at P1 did *not* result in altered densities. Comparing the results following SC vehicle injections at P1 and P5, averaged E15 RGC densities were significantly reduced from 2,938 ± 143 RGCs/mm^2^ to 1,518 ± 288 RGCs/mm^2^, respectively (~26% reduction in estimated numbers), and this was further decreased to 787 ± 18 RGCs/mm^2^ after blocking injections (~50% further reduction; Figure 6A).

The proportion of E15 RGCs quantified as a percentage of total RGCs in the ganglion cell layer was significantly greater after P1 retinal injections compared to P1 and P5 SC injections [*F*(2, 20) = 5.56, *p* < 0.05; Sidak, *p* < 0.05; Figure 6B]. A main effect of blocking antibody injections [*F*(1, 20) = 6.03, *p* < 0.05] suggests that this treatment reduced the percentage of E15 RGCs compared to vehicle injections, however, the lack of a significant interaction effect [*F*(2, 20) = 1.55, *p* = 0.24] prevented any further interpretation of whether this decrease occurred in all groups, or only with SC injections. In sum, these results demonstrate an overall reduction in E15 RGC density and numbers during early postnatal development, and that injections of combined BDNF and NT-4/5 blocking antibodies into the SC but *not* into the retina increases this loss. The data confirm the effectiveness of the BDNF and NT-4/5 blocking antibodies and show that, in the early postnatal period, early-born RGCs with axons already in visual centers depend primarily on target derived trophic support.

### *In vivo* E18 RGCs

Similar to the *in vitro* analysis comparing different aged RGCs, the number of E18 RGCs after P1 and P5 injections was considerably less when compared to E15 RGCs. Unlike E15 RGCs, the density of E18 RGCs did not significantly differ with age [*F*(3, 29) = 2.62, *p* = 0.07], nor after blocking injections into the eye or SC when compared to controls [*F*(1, 29) = 3.12, *p* = 0.09; Figure 6C]. However, the percentage of E18 RGCs as a proportion of the total RGC population significantly increased with age [*F*(3, 29) = 8.71, *p* < 0.01; Sidak < 0.01; Figure 6D]. Combining retinal and SC vehicle injection data (Figure 6C), the density of E18 RGCs averaged 520 ± 84 RGCs/mm^2^ at P2 and 361 ± 56 RGCs/mm^2^at P6. This equated to approximately 18% and 24% of the averaged (combined retina and SC vehicle data) E15 density at P2 and P6, suggesting that the increase in the percentage of E18 RGCs is reflective of the more extensive loss of a large number of earlier born RGCs.

## Discussion

The aim of the study was to investigate neurotrophin dependency in RGC cohorts at different developmental ages and relate this to the timing of innervation of central visual targets in the rat midbrain. RGCs were pre-labeled with BrdU at two different embryonic ages, and using *in vitro* and *in vivo* methods, we tested the impact of two different approaches previously demonstrated to reduce neurotrophin activation of TrkB receptors. There was a significant reduction in the number of P1 purified E15 RGCs when cultured in the absence of exogenous BDNF, but addition of recombinant BDNF to the cultures maintained E15 RGC viability for at least 48 h (see also Moses et al., 2015). In contrast, E18 RGC numbers remained stable over 48 h in culture even when exogenous BDNF was absent; however, in this condition, there was a trend for reduced viability when BDNF and NT-4/5 antibodies or TrkB-Fc were added to the media, presumably by reducing at least some TrkB activity. *In vivo*, depleting TrkB neurotrophins in the SC, but *not* in the retina, at both P1 and P5 reduced the percentage and density of E15 RGCs; however, injections of the same blocking antibodies in either location at either P1 or P5 did not measurably affect E18 RGC densities.

### Combined Cultures and *in vivo* Counts—Irrespective of Birthdate

A general trend in the *in vitro* studies was that the various treatments did not affect overall RGC survival, independent of birthdate; at 48 h all groups demonstrated a similar proportion of surviving RGCs relative to initial seeding numbers. In the case of BDNF, these data differ from previous studies using purified RGCs in which addition of this neurotrophin increased overall RGC viability (Johnson et al., 1986; Meyer-Franke et al., 1995; Moses et al., 2015). Nonetheless, exogenous BDNF application clearly improved the survival of E15 RGCs and facilitated the elaboration of complex processes, similar to that reported previously (Johnson et al., 1986; Bosco and Linden, 1999; Marler et al., 2014; Moses et al., 2015), establishing the efficacy of our BDNF treatment. The observed differences in overall RGC survival may be due to differing seeding densities between studies, which may affect the availability of exogenous BDNF, or subtle differences in the age of RGCs at the time of plating.

Surprisingly, unlike previous studies in our lab which have shown that depletion of TrkB ligands *in vivo* induces RGC death (Spalding et al., 2004), we did not witness a reduction in overall RGC survival *in vitro* with BDNF and NT-4/5 antibodies. While at first glance this may indicate that our blocking methods may not have been adequate, these same blocking antibodies reduced BDNF-induced neurite elaboration *in vitro*, and reduced the number and density of E15 RGCs both in culture and when injected into the SC (Figures 3B, 6A). These findings suggest that the blocking antibodies were effective in reducing the action of TrkB, as has also been found in previously published experiments: similar BDNF and NT4-5 antibodies have been used in many *in vitro* and *in vivo* studies to deplete neurotrophin availability, and have been verified to reduce downstream signaling of TrkB in primary neuronal cultures (Lepack et al., 2016), reduce neurotrophin reactivity (Zhou and Rush, 1996), reduce TrkB phosphorylation in the rat visual cortex (Jiang et al., 2003), reduce RGC terminal arbor complexity *in vivo* (Cohen-Cory, 1999), and prevent other BDNF induced functional changes such as physiology and behavior (Balkowiec and Katz, 2000; Graham et al., 2007; Peters et al., 2010; Rosas-Vidal et al., 2014; Lepack et al., 2015).

A possible reason for the lack of effect of neurotrophin depletion on total RGC survival *in vitro* may be that the concentration of blocking antibodies, or the TrkB-Fc chimera, was not high enough (in molar excess) to inhibit all local neurotrophin activity, especially when combined with exogenous BDNF for a 48 h period. In addition, it is possible that at least some RGC viability was supported by other factors, including other neurotrophins (Argaw et al., 2008; Braunger et al., 2013). For example, neurotrophin-3 (NT-3) is produced by RGCs and can act on the TrkB receptor, albeit at lower efficacy (Barbacid, 1994; Huang et al., 1999; Agarwal et al., 2007). Although NT-3 does not appear to have a major impact on the survival of immature RGCs (Cui and Harvey, 1995) or neurite outgrowth (Atkinson et al., 1999; Bosco and Linden, 1999), NT-3 mRNA has been shown to be upregulated following RGC injury in chicks, perhaps relevant to this this study given that the majority of purified RGCs were axotomized and dissociated from their usual tissue support (De la Rosa et al., 1994).

*In vivo*, a decrease in overall RGC density was seen with blocking antibody injections into the SC at P1 but not at P5. Previous studies have demonstrated that peak RGC death occurs around P1–P4 when early-born RGCs (E13–E16) have axons in the SC target (Potts et al., 1982; Dreher et al., 1983). Addition of blocking neurotrophins in this central target location may therefore exacerbate the magnitude of death by further reducing the availability of neurotrophins at a time when competition between a large population of ingrowing RGC axons is greatest.

Overall, there was an approximate 50% decrease in Brn3a^+^ RGC density seen *in vivo* between P2 and P6, consistent with previous studies showing extensive RGC death during this period (Jeffery and Perry, 1981; Dreher et al., 1983; Sefton and Lam, 1984; Young, 1984; O’Leary et al., 1986; Beros et al., 2018) and developmental loss of Brn3a^+^ labeled RGCs in retinal sections (Voyatzis et al., 2012). However, this observed decrease in RGC density may also have been influenced to a lesser degree by postnatal expansion of the retina during this same period. Our estimates of retinal surface area are lower than those previously reported (McCall et al., 1987), perhaps due to shrinkage during fixation, the use of HCl during BrdU immunohistochemistry, and estimation errors when obtaining surface areas from retinal sections (that do not include the most peripheral parts of retina). The surface area of the assessed retinas was approximately 27% larger in the P5 injection groups compared to P1. If retinal growth was the only factor causing the density changes at these ages and not cell death, we would expect a reduction in density mirroring the difference in size from P2 to P6 (a 27% reduction). However, our obtained densities at P6 are consistently lower than this value, indicative of the additional, cumulative impact of naturally occurring RGC death.

### Neurotrophin Depletion and Survival of E15 and E18 RGC Cohorts

Similar to previous studies, we observed not only a greater amount of RGCs born at E15 than at E18 (Reese and Colello, 1992; Rapaport et al., 2004), but that far more early-born RGCs are lost in the first few days following birth. In addition, we show that RGCs born early (E15) or late (E18) displayed clear age-specific responses to neurotrophins and their depletion. Our findings confirm previous results showing that, in neonatal cultures, E15 RGCs require exogenous BDNF for prolonged survival (Moses et al., 2015), and add the novel *in vivo* finding that selective neurotrophin depletion, specifically in the SC, significantly increases the loss of identified E15 RGCs. Increased RGC pyknotic profiles 24 h but not 48 h after injection of blocking antibodies into the SC at P4 has been reported previously (Spalding et al., 2004) but in that earlier study the birthdate of the RGCs was not characterized. These neurotrophins are normally produced by cells within the neonatal SC (Frost et al., 2001; Marotte et al., 2004) and our new data are consistent with the increased RGC death that is seen after injection of kainic acid into the SC, a method of ablation that kills target neurons but does not damage RGC axons (Carpenter et al., 1986; Spalding et al., 2004). Most importantly, and consistent with the target switching hypothesis (Moses et al., 2015), injection of blocking antibodies into the eye at P1 or P5 did *not* elicit a significant reduction in the density of surviving E15 RGCs compared to vehicle injections.

In contrast to the E15 RGC *in vitro* data, the viability of cultured E18 RGCs isolated at P1 was not dependent on the presence of exogenous BDNF. Unlike E15 RGCs, which have axons present in the SC before birth and are dependent upon target secreted neurotrophins from at least P0 onward, E18 RGCs are clearly viable, yet do not have axons in the SC at that early age Dallimore et al., 2002). E18 RGCs isolated from retinal tissue just after birth express high levels of mRNA encoding BDNF as well as signaling proteins downstream of TrkB involved in cell survival (Moses et al., 2015), suggesting that these cells produce their own source of BDNF and pro-survival factors that can also support nearby RGCs via paracrine mechanisms (De Araujo and Linden, 1993; Perez and Caminos, 1995; Cellerino and Kohler, 1997; Moses et al., 2015). Consistent with this, in the rat, E18/19 RGCs *in vivo* do not begin to die until P5/6, shortly after the arrival of their axons in the SC (Dallimore et al., 2010), and at the time endogenous RGC expression of BDNF mRNA is reported to be reduced (Moses et al., 2015).

E18 RGCs responded differently to blocking neurotrophins both *in vitro* and *in vivo*. In the absence of exogenous BDNF *in vitro*, blocking antibodies applied to culture wells resulted in an approximate 25–40% reduction in E18 RGCs at 24 and 48 h, and a similar trend of decreased viability was also seen after application of the BDNF scavenger TrkB-Fc. The timing of this reduction is consistent with autocrine and paracrine expression of BDNF in these late-born neurons (Moses et al., 2015). However, despite quantitative analysis of nearly 100 culture wells, this reduction was not found to be significant, likely due to the low E18 cell counts and associated variance between biological replicates. An additional source of variation is the timing of mating and birth of pups. Dams were mated overnight but the exact timing of fertilization is unknown and litters could be born over a period of several hours during the day designated as P0. Both of these factors would increase variation in the small numbers of E18 RGCs more than in the much larger population of RGCs born on E15. *In vivo*, depletion of neurotrophins in the retina or SC also did not significantly reduce E18 RGC densities when injected at either P1 or P5. Again, E18 RGC counts were low and variance high. Note that a previous study did report that intravitreal injection of BDNF and NT-4/5 blocking antibodies temporarily increased neonatal RGC pyknosis, but these RGCs were not birthdated (Spalding et al., 2004). There was a significant increase in the percentage of E18 RGCs quantified at P6 compared to P2, likely a consequence of the death of a great many earlier born RGCs resulting in a proportionally greater representation of late-born RGCs.

Our data show that the response of RGCs to BDNF is age specific and suggest that there are differences in the factors that influence death in the early versus late-born cohorts. Although not directly assessed in our study, competition for target-derived trophic support by developing neurons is an established phenomenon (Purves, 1988; Oppenheim, 1991) and E15 RGCs presumably compete for factors such as BDNF during innervation of central targets (Moses et al., 2015), although such death does not appear to be linked to initial topographic mapping errors (Voyatzis et al., 2012; Beros et al., 2018). On the other hand, the smaller number of late-born RGCs may behave differently: in mice such neurons are known to be more accurate in their target selection (Osterhout et al., 2014), their axons reach a more mature SC much later (Dallimore et al., 2002), and they may be less dependent on competition to drive target selection. Later-born RGCs with a more protracted axon growth phase (Dallimore et al., 2010) may also have more opportunity to encounter a wide range of factors reported to positively impact RGC viability (Rehen et al., 2002; Argaw et al., 2008; Ma and Taylor, 2010; Corredor et al., 2012; Moses et al., 2015). Interestingly then, while the survival of injured adult RGCs can generally be enhanced by intraocular injection of recombinant BDNF, or after injection of vectors encoding the BDNF gene (Harvey et al., 2012), birth-dated E18/19 RGCs are more likely than E15 RGCs to survive for a prolonged period in rats with a peripheral nerve grafted onto the transected optic nerve (Dallimore et al., 2010).

In addition, there may be differences in the sensitivity of late-born RGC cohorts toward target derived neurotrophic factors during development. Axotomy/target ablation within the first postnatal week results in rapid RGC death that peaks at 24 h and is mostly resolved by 48 h (Harvey and Robertson, 1992; Spalding et al., 2004). Beginning from P7, this sensitivity is noticeably reduced in the rat, as kainic acid injections into the SC results in the death of fewer RGCs when compared to injections at P3–4 (Carpenter et al., 1986), even though BDNF levels (measured in hamster SC) remain higher than adult levels until about P14 (Frost et al., 2001). In the adult, axotomy induced RGC death is slower, peaking at 7 days post-lesion and continuing for several weeks (Villegas-Pérez et al., 1993; Berkelaar et al., 1994; Isenmann et al., 1997; Klöcker et al., 1998). Although the effects of SC injections of BDNF have not been analyzed in relation to E15 and E18 RGCs, these different aged cohorts do have increased sensitivity to the removal of target derived neurotrophic factors (via SC ablation), especially during the period when their axons are growing into and innervating the SC (Dallimore et al., 2010). Compared to E15 RGCs where death was observed in our *in vivo* study at 24 h post blocking injection, E18 RGCs may undergo a more prolonged period of death and the cumulative effects may not be observable until after this period. In addition, E18 RGC axons are known to arrive in the SC at about P5, but a case may be made that not all E18 RGCs have their axons present in the SC at this age, because previous studies assessing E18 RGC target innervation and gene expression have not investigated times later than P6 (Dallimore et al., 2002, 2010; Moses et al., 2015). Of relevance is the finding that from P1 to P5, E18 RGC gene expression patterns become increasingly similar to, but not the same as their E15 counterparts at P1 (whose axons have been present in the SC for a longer period of time at testing) (Moses et al., 2015). Thus, P5-P6 may reflect a transitionary time, as this cohort may contain a mix of RGCs that have or have not yet reached the SC and the switch to target derived neurotrophic dependence may occur over a prolonged period as axons arrive *de novo*.

Consideration must also be made to the many subtypes that make up the RGC population, given that they can be generated at different developmental days (Reese and Colello, 1992; Osterhout et al., 2014). Our lab has shown that RGC birthdate influences the post-injury survival and regenerative ability of adult rat RGCs after optic nerve injury (Dallimore et al., 2010), and recent evidence in the mouse has reported that such responses may vary by RGC subtype (Tran et al., 2019). Tran et al. (2019) performed RNA sequencing on RGCs after an optic nerve crush and observed differential resilience between 46 identified RGC subtypes, accompanied with differences in gene expression. If RGC subtypes respond differently in their response to physical injury, then this might translate to differences in their response to the manipulations in our study. If the more resilient cells were part of the later E18 cohort for example, then this might help in understanding why blocking TrkB activity did not elicit a measurable effect on survival, both *in vitro* and *in vivo.* Although while we acknowledge that the aged cohorts in our study may consist of different RGC subtypes, Moses et al. (2015) demonstrated that, irrespective of subtype, gene expression patterns of E18 RGCs differed between P1 and P5, and by P5 become increasingly similar to the expression of target dependent E15 RGCs assessed at P1. As the main difference exhibited by the E18 cohort over time is increased maturation and axonal growth, this suggests that the age of RGCs and the timing of target innervation is a consistent predictor of their dependence on BDNF during development. Note here that differential sensitivity to trophic support may also be related to the observation that E15 RGCs possess axons that innervate central targets within perhaps 3 days after cell birth, whereas axons from late-born (E19) RGCs require 8–9 days to reach their now more mature and complex targets. Thus these later-born neurons must survive independent of central trophic support for a more protracted period (Dallimore et al., 2002, 2010).

## Conclusion

Our study supports previous literature demonstrating that in rats, far fewer RGCs are born at E18 compared to E15, and that more early-born RGCs are eliminated in the first few days following birth. We also present new data demonstrating age-related responses of RGCs to the presence of neurotrophic factors during the early postnatal period, effects that are evident both *in vitro* and *in vivo*. Cultured E15 RGCs require exogenous BDNF for sustained viability, and *in vivo* these early-born RGCs with axons already in the SC at birth specifically require target derived BDNF and the action of its TrkB receptor for their continued survival and maturation. In contrast, late-born RGCs do not require exogenous BDNF for their survival for 48 h *in vitro*, and there was no measurable effect resulting from depletion of BDNF (and NT-4/5) in either the eye or SC during the first postnatal week. Together, these results suggest that neuronal birthdate and post-mitotic age, the timing of target innervation, and neurotrophin dependence are integrated into a complex process of retinotectal innervation and map formation. The data also suggest that the mechanisms associated with RGC death may differ depending on birthdate: later-born RGCs that grow for a more prolonged period in a more differentiated environment before they reach central targets may be influenced by a broader range of supportive factors that aid their survival and maturation (Rehen et al., 2002; Argaw et al., 2008; Ma and Taylor, 2010; Corredor et al., 2012; Moses et al., 2015).

## Data Availability Statement

The raw data supporting the conclusions of this article will be made available by the authors, without undue reservation.

## Ethics Statement

The animal study was reviewed and approved by the UWA Animal Ethics Committee.

## Author Contributions

JB, JR, and AH contributed to conception and design of the study. JB performed experimental work, analysis, and wrote the first draft of the manuscript. All authors contributed to the manuscript revision, read, and approved the submitted version.

## Conflict of Interest

The authors declare that the research was conducted in the absence of any commercial or financial relationships that could be construed as a potential conflict of interest.

## References

[B1] Agarwal N. Agarwal R. Kumar D. M. Ondricek A. Clark A. F. Wordinger R. J. (2007). Comparison of expression profile of neurotrophins and their receptors in primary and transformed rat retinal ganglion cells. *Mol. Vis.* 13 1311–1318. 17679933

[B2] Ahmed A. F. Dong K. Setsu T. Yamadori T. (1996). Correlation between different types of retinal ganglion cells and their projection pattern in the albino rat. *Brain Res.* 706 163–168. 10.1016/0006-8993(95)01283-4 8720506

[B3] Argaw A. Duff G. Boire D. Ptito M. Bouchard J. F. (2008). Protein kinase A modulates retinal ganglion cell growth during development. *Exp. Neurol.* 211 494–502. 10.1016/j.expneurol.2008.02.014 18423622

[B4] Atkinson J. Panni M. K. Lund R. D. (1999). Effects of neurotrophins on embryonic retinal outgrowth. *Dev. Brain Res.* 112 173–180. 10.1016/s0165-3806(98)00165-5 9878724

[B5] Balkowiec A. Katz D. M. (2000). Activity-dependent release of endogenous brain-derived neurotrophic factor from primary sensory neurons detected by ELISA in situ. *J. Neurosci.* 20 7417–23. 10.1523/jneurosci.20-19-07417.2000 11007900 PMC6772775

[B6] Barbacid M. (1994). The Trk family of neurotrophin receptors. *J. Neurobiol.* 25 1386–1403. 10.1002/neu.480251107 7852993

[B7] Berkelaar M. Clarke D. Wang Y. Bray G. Aguayo A. (1994). Axotomy results in delayed death and apoptosis of retinal ganglion cells in adult rats. *J. Neurosci.* 14 4368–4374. 10.1523/jneurosci.14-07-04368.1994 8027784 PMC6577016

[B8] Beros J. Rodger J. Harvey A. R. (2018). Developmental retinal ganglion cell death and retinotopicity of the murine retinocollicular projection. *Dev. Neurobiol.* 78 51–60. 10.1002/dneu.22559 29134765

[B9] Bosco A. Linden R. (1999). BDNF and NT-4 differentially modulate neurite outgrowth in developing retinal ganglion cells. *J. Neurosci. Res.* 57 759–769. 10.1002/(sici)1097-4547(19990915)57:6<759::aid-jnr1>3.0.co;2-y 10467247

[B10] Braunger B. M. Pielmeier S. Demmer C. Landstorfer V. Kawall D. Abramov N. (2013). TGF-β signaling protects retinal neurons from programmed cell death during the development of the mammalian eye. *J. Neurosci.* 33 14246–14258. 10.1523/jneurosci.0991-13.2013 23986258 PMC6618509

[B11] Breuss M. W. Leca I. Gstrein T. Hansen A. H. Keays D. A. (2017). Tubulins and brain development–The origins of functional specification. *Mol. Cell. Neurosci.* 84 58–67. 10.1016/j.mcn.2017.03.002 28347630

[B12] Bunt S. M. Lund R. Land P. (1983). Prenatal development of the optic projection in albino and hooded rats. *Dev. Brain Res.* 6 149–168. 10.1016/0165-3806(83)90093-7 6831237

[B13] Carpenter P. Sefton A. J. Dreher B. Lim W. L. (1986). Role of target tissue in regulating the development of retinal ganglion cells in the albino rat: effects of kainate lesions in the superior colliculus. *J. Comp. Neurol.* 251 240–259. 10.1002/cne.902510208 3782500

[B14] Cellerino A. Kohler K. (1997). Brain-derived neurotrophic factor/neurotrophin-4 receptor TrkB is localized on ganglion cells and dopaminergic amacrine cells in the vertebrate retina. *J. Comp. Neurol.* 386 149–160. 10.1002/(sici)1096-9861(19970915)386:1<149::aid-cne13>3.0.co;2-f 9303531

[B15] Cohen A. Bray G. Aguayo A. (1994). Neurotrophin-4/5 (NT-4/5) increases adult rat retinal ganglion cell survival and neurite outgrowth in vitro. *J. Neurobiol.* 25 953–959. 10.1002/neu.480250805 7964706

[B16] Cohen-Cory S. (1999). BDNF modulates, but does not mediate, activity-dependent branching and remodeling of optic axon arbors in vivo. *J. Neurosci.* 19 9996–10003. 10.1523/jneurosci.19-22-09996.1999 10559407 PMC6782987

[B17] Corredor R. G. Trakhtenberg E. F. Pita-Thomas W. Jin X. Hu Y. Goldberg J. L. (2012). Soluble adenylyl cyclase activity is necessary for retinal ganglion cell survival and axon growth. *J. Neurosci.* 32 7734–7744. 10.1523/jneurosci.5288-11.2012 22649251 PMC3372574

[B18] Coull J. A. M. Beggs S. Boudreau D. Boivin D. Tsuda M. Inoue K. (2005). BDNF from microglia causes the shift in neuronal anion gradient underlying neuropathic pain. *Nature* 438 1017–1021. 10.1038/nature04223 16355225

[B19] Crespo D. O’Leary D. D. Cowan W. M. (1985). Changes in the numbers of optic nerve fibers during late prenatal and postnatal development in the albino rat. *Dev. Brain Res.* 19 129–134. 10.1016/0165-3806(85)90238-x 3995334

[B20] Cui Q. Harvey A. (1995). At least two mechanisms are involved in the death of retinal ganglion cells following target ablation in neonatal rats. *J. Neurosci.* 15 8143–8155. 10.1523/jneurosci.15-12-08143.1995 8613749 PMC6577937

[B21] Dallimore E. J. Cui Q. Beazley L. D. Harvey A. R. (2002). Postnatal innervation of the rat superior colliculus by axons of late-born retinal ganglion cells. *Eur. J. Neurosci.* 16 1295–1304. 10.1046/j.1460-9568.2002.02178.x 12405990

[B22] Dallimore E. J. Park K. K. Pollett M. A. Taylor J. S. Harvey A. R. (2010). The life, death and regenerative ability of immature and mature rat retinal ganglion cells are influenced by their birthdate. *Exp. Neurol.* 225 353–365. 10.1016/j.expneurol.2010.07.007 20643130

[B23] Davies A. M. (1994). The role of neurotrophins in the developing nervous system. *J. Neurobiol.* 25 1334–1348. 10.1002/neu.480251103 7852989

[B24] Davies A. M. (1996). The neurotrophic hypothesis: where does it stand? *Philos. Trans. R. Soc. Lond. B Biol. Sci.* 351 389–394. 10.1098/rstb.1996.0033 8730776

[B25] De Araujo E. G. Linden R. (1993). Trophic factors produced by retinal cells increase the survival of retinal ganglion cells in vitro. *Eur. J. Neurosci.* 5 1181–1188. 10.1111/j.1460-9568.1993.tb00972.x 8281322

[B26] De la Rosa E. Arribas A. Frade J. Rodri A. (1994). Role of neurotrophins in the control of neural development: neurotrophin-3 promotes both neuron differentiation and survival of cultured chick retinal cells. *Neuroscience* 58 347–352. 10.1016/0306-4522(94)90041-8 8152543

[B27] Dreher B. Potts R. Bennett M. (1983). Evidence that the early postnatal reduction in the number of rat retinal ganglion cells is due to a wave of ganglion cell death. *Neurosci. Lett.* 36 255–260. 10.1016/0304-3940(83)90009-5 6866331

[B28] Ernfors P. Merlio J. P. Persson H. (1992). Cells expressing mRNA for neurotrophins and their receptors during embryonic rat development. *Eur. J. Neurosci.* 4 1140–1158. 10.1111/j.1460-9568.1992.tb00141.x 12106420

[B29] Frost D. O. Ma Y. T. Hsieh T. Forbes M. E. Johnson J. E. (2001). Developmental changes in BDNF protein levels in the hamster retina and superior colliculus. *Dev. Neurobiol.* 49 173–187. 10.1002/neu.1073 11745656

[B30] Ghosh A. Carnahan J. Greenberg M. E. (1994). Requirement for BDNF in activity-dependent survival of cortical neurons. *Science* 263 1618–23. 10.1126/science.7907431 7907431

[B31] Graham D. L. Edwards S. Bachtell R. K. DiLeone R. J. Rios M. Self D. W. (2007). Dynamic BDNF activity in nucleus accumbens with cocaine use increases self-administration and relapse. *Nat. Neurosci.* 10 1029–1037. 10.1038/nn1929 17618281

[B32] Harvey A. R. Ooi J. W. W. Rodger J. (2012). Chapter one - neurotrophic factors and the regeneration of adult retinal ganglion cell axons. *Int. Rev. Neurobiol.* 106 1–33. 10.1016/b978-0-12-407178-0.00002-8 23211458

[B33] Harvey A. R. Robertson D. (1992). Time-course and extent of retinal ganglion cell death following ablation of the superior colliculus in neonatal rats. *J. Comp. Neurol.* 325 83–94. 10.1002/cne.903250108 1484120

[B34] Huang E. J. Reichardt L. F. (2001). Neurotrophins: roles in neuronal development and function. *Annu. Rev. Neurosci.* 24 677–736. 10.1146/annurev.neuro.24.1.677 11520916 PMC2758233

[B35] Huang E. J. Wilkinson G. A. Fariñas I. Backus C. Zang K. Wong S. L. (1999). Expression of Trk receptors in the developing mouse trigeminal ganglion: in vivo evidence for NT-3 activation of TrkA and TrkB in addition to TrkC. *Development* 126 2191–2203. 10.1242/dev.126.10.2191 10207144 PMC2710120

[B36] Isenmann S. Wahl C. Krajewski S. Reed J. C. Bähr M. (1997). Up-regulation of Bax protein in degenerating retinal ganglion cells precedes apoptotic cell death after optic nerve lesion in the rat. *Eur. J. Neurosci.* 9 1763–1772. 10.1111/j.1460-9568.1997.tb01534.x 9283831

[B37] Jeffery G. Perry V. (1981). Evidence for ganglion cell death during development of the ipsilateral retinal projection in the rat. *Dev. Brain Res.* 2 176–180. 10.1016/0165-3806(81)90069-9 7272770

[B38] Jelsma T. N. Friedman H. H. Berkelaar M. Bray G. M. Aguayo A. J. (1993). Different forms of the neurotrophin receptor trkB mRNA predominate in rat retina and optic nerve. *J. Neurobiol.* 24 1207–1214. 10.1002/neu.480240907 8409978

[B39] Jiang B. Akaneya Y. Hata Y. Tsumoto T. (2003). Long-term depression is not induced by low-frequency stimulation in rat visual cortex in vivo: a possible preventing role of endogenous brain-derived neurotrophic factor. *J. Neurosci.* 23 3761–70. 10.1523/jneurosci.23-09-03761.2003 12736347 PMC6742196

[B40] Johnson J. E. Barde Y.-A. Schwab M. Thoenen H. (1986). Brain-derived neurotrophic factor supports the survival of cultured rat retinal ganglion cells. *J. Neurosci.* 6 3031–3038. 10.1523/jneurosci.06-10-03031.1986 2876066 PMC6568792

[B41] Jose A. Soriano E. (1989). Immunocytochemical detection of 5′-bromodeoxyuridine incorporation in the central nervous system of the mouse. *Dev. Brain Res.* 49 311–317. 10.1016/0165-3806(89)90033-3 2805336

[B42] Klöcker N. Cellerino A. Bähr M. (1998). Free radical scavenging and inhibition of nitric oxide synthase potentiates the neurotrophic effects of brain-derived neurotrophic factor on axotomized retinal ganglion cells in vivo. *J. Neurosci.* 18 1038–1046. 10.1523/jneurosci.18-03-01038.1998 9437024 PMC6792783

[B43] Koide T. Takahashi J. B. Hoshimaru M. Kojima M. Otsuka T. Asahi M. (1995). Localization of trkB and low-affinity nerve growth factor receptor mRNA in the developing rat retina. *Neurosci. Lett.* 185 183–186. 10.1016/0304-3940(95)11257-w 7753487

[B44] Langer T. Lund R. (1974). The upper layers of the superior colliculus of the rat: a Golgi study. *J. Comp. Neurol.* 158 405–435. 10.1002/cne.901580404 4615112

[B45] Lepack A. E. Bang E. Lee B. Dwyer J. M. Duman R. S. (2016). Fast-acting antidepressants rapidly stimulate ERK signaling and BDNF release in primary neuronal cultures. *Neuropharmacology* 111 242–252. 10.1016/j.neuropharm.2016.09.011 27634096 PMC5075989

[B46] Lepack A. E. Fuchikami M. Dwyer J. M. Banasr M. Duman R. S. (2015). BDNF release is required for the behavioral actions of ketamine. *Int. J. Neuropsychopharmacol.* 18:pyu033. 10.1093/ijnp/pyu033 25539510 PMC4368871

[B47] Linden R. Perry V. (1983). Massive retinotectal projection in rats. *Brain Res.* 272 145–149. 10.1016/0006-8993(83)90371-2 6616190

[B48] Lund R. Land P. Boles J. (1980). Normal and abnormal uncrossed retinotectal pathways in rats: an HRP study in adults. *J. Comp. Neurol.* 189 711–720. 10.1002/cne.901890407 7381047

[B49] Ma C. H. E. Taylor J. S. (2010). Trophic responsiveness of purified postnatal and adult rat retinal ganglion cells. *Cell Tissue Res.* 339 297–310. 10.1007/s00441-009-0897-4 19936794

[B50] Ma Y.-T. Hsieh T. Forbes M. E. Johnson J. E. Frost D. O. (1998). BDNF injected into the superior colliculus reduces developmental retinal ganglion cell death. *J. Neurosci.* 18 2097–2107. 10.1523/jneurosci.18-06-02097.1998 9482796 PMC6792912

[B51] Mariga A. Zavadil J. Ginsberg S. D. Chao M. V. (2015). Withdrawal of BDNF from hippocampal cultures leads to changes in genes involved in synaptic function. *Dev. Neurobiol.* 75 173–192. 10.1002/dneu.22216 25059794 PMC4329925

[B52] Marler K. J. Poopalasundaram S. Broom E. R. Wentzel C. Drescher U. (2010). Pro-neurotrophins secreted from retinal ganglion cell axons are necessary for ephrinA-p75NTR-mediated axon guidance. *Neural Dev.* 5:30. 10.1186/1749-8104-5-30 21044296 PMC2987844

[B53] Marler K. J. Suetterlin P. Dopplapudi A. Rubikaite A. Adnan J. Maiorano N. A. (2014). BDNF promotes axon branching of retinal ganglion cells via miRNA-132 and p250GAP. *J. Neurosci.* 34 969–979. 10.1523/jneurosci.1910-13.2014 24431455 PMC3891972

[B54] Marotte L. Vidovic M. Wheeler E. Jhaveri S. (2004). Brain-derived neurotrophic factor is expressed in a gradient in the superior colliculus during development of the retinocollicular projection. *Eur. J. Neurosci.* 20 843–847. 10.1111/j.1460-9568.2004.03521.x 15255995

[B55] McCall M. J. Robinson S. R. Dreher B. (1987). Differential retinal growth appears to be the primary factor producing the ganglion cell density gradient in the rat. *Neurosci. Lett.* 79 78–84. 10.1016/0304-3940(87)90675-6 3670734

[B56] McLaughlin T. Torborg C. L. Feller M. B. O’Leary D. D. (2003). Retinotopic map refinement requires spontaneous retinal waves during a brief critical period of development. *Neuron* 40 1147–1160. 10.1016/s0896-6273(03)00790-6 14687549

[B57] Meyer-Franke A. Kaplan M. R. Pfieger F. W. Barres B. A. (1995). Characterization of the signaling interactions that promote the survival and growth of developing retinal ganglion cells in culture. *Neuron* 15 805–819. 10.1016/0896-6273(95)90172-8 7576630

[B58] Moses C. Wheeler L. P. LeVaillant C. J. Kramer A. Ryan M. Cozens G. S. (2015). The acquisition of target dependence by developing rat retinal ganglion cells. *eNeuro* 2 1–20. 10.1523/ENEURO.0044-14.2015 PMC4586937 26464991

[B59] Nadal-Nicolás F. M. Jiménez-López M. Sobrado-Calvo P. Nieto-López L. Cánovas-Martínez I. Salinas-Navarro M. (2009). Brn3a as a marker of retinal ganglion cells: qualitative and quantitative time course studies in naive and optic nerve–injured retinas. *Invest. Ophthalmol. Vis. Sci.* 50 3860–3868. 10.1167/iovs.08-3267 19264888

[B60] Nagappan G. Lu B. (2005). Activity-dependent modulation of the BDNF receptor TrkB: mechanisms and implications. *Trends Neurosci.* 28 464–471. 10.1016/j.tins.2005.07.003 16040136

[B61] O’Leary D. Fawcett J. W. Cowan W. M. (1986). Topographic targeting errors in the retinocollicular projection and their elimination by selective ganglion cell death. *J. Neurosci.* 6 3692–3705. 10.1523/jneurosci.06-12-03692.1986 3794796 PMC6568660

[B62] Oppenheim R. W. (1991). Cell death during development of the nervous system. *Annu. Rev. Neurosci.* 14 453–501. 10.1146/annurev.ne.14.030191.002321 2031577

[B63] Osterhout J. A. El-Danaf R. N. Nguyen P. L. Huberman A. D. (2014). Birthdate and outgrowth timing predict cellular mechanisms of axon target matching in the developing visual pathway. *Cell Rep.* 8 1006–1017. 10.1016/j.celrep.2014.06.063 25088424 PMC4143387

[B64] Packard D. Jr. Menzies R. Skalko R. (1973). Incorporation of thymidine and its analogue, bromodeoxyuridine, into embryos and maternal tissues of the mouse. *Differentiation* 1 397–405. 10.1111/j.1432-0436.1973.tb00137.x 4802502

[B65] Perez M.-T. R. Caminos E. (1995). Expression of brain-derived neurotrophic factor and of its functional receptor in neonatal and adult rat retina. *Neurosci. Lett.* 183 96–99. 10.1016/0304-3940(94)11123-z 7746496

[B66] Perry V. Henderson Z. Linden R. (1983). Postnatal changes in retinal ganglion cell and optic axon populations in the pigmented rat. *J. Comp. Neurol.* 219 356–368. 10.1002/cne.902190309 6619343

[B67] Peters J. Dieppa-Perea L. M. Melendez L. M. Quirk G. J. (2010). Induction of fear extinction with hippocampal-infralimbic BDNF. *Science* 328 1288–90. 10.1126/science.1186909 20522777 PMC3570764

[B68] Pimentel B. Sanz C. Varela-Nieto I. Rapp U. R. De Pablo F. Enrique J. (2000). c-Raf regulates cell survival and retinal ganglion cell morphogenesis during neurogenesis. *J. Neurosci.* 20 3254–3262. 10.1523/jneurosci.20-09-03254.2000 10777790 PMC6773115

[B69] Potts R. Dreher B. Bennett M. (1982). The loss of ganglion cells in the developing retina of the rat. *Dev. Brain Res.* 3 481–486. 10.1016/0165-3806(82)90013-x 7066701

[B70] Purves D. (1988). *Body and Brain: A Trophic Theory of Neural Connections.* Cambridge: Harvard University Press. 10.1126/science.244.4907.993 17731884

[B71] Quina L. A. Pak W. Lanier J. Banwait P. Gratwick K. Liu Y. (2005). Brn3a-expressing retinal ganglion cells project specifically to thalamocortical and collicular visual pathways. *J. Neurosci.* 25 11595–11604. 10.1523/jneurosci.2837-05.2005 16354917 PMC6726022

[B72] Rapaport D. H. Wong L. L. Wood E. D. Yasumura D. LaVail M. M. (2004). Timing and topography of cell genesis in the rat retina. *J. Comp. Neurol.* 474 304–324. 10.1002/cne.20134 15164429

[B73] Reese B. Colello R. (1992). Neurogenesis in the retinal ganglion cell layer of the rat. *Neuroscience* 46 419–429. 10.1016/0306-4522(92)90062-7 1542415

[B74] Rehen S. K. Cid M. Fragel-Madeira L. Linden R. (2002). Differential effects of cyclin-dependent kinase blockers upon cell death in the developing retina. *Brain Res.* 947 78–83. 10.1016/s0006-8993(02)02909-8 12144855

[B75] Rosas-Vidal L. E. Do-Monte F. H. Sotres-Bayon F. Quirk G. J. (2014). Hippocampal–prefrontal BDNF and memory for fear extinction. *Neuropsychopharmacology* 39 2161–2169. 10.1038/npp.2014.64 24625752 PMC4104333

[B76] Rosenthal A. Lin J. C. (2014). “Modulation of neurotrophin signaling by monoclonal antibodies,” in *Neurotrophic Factors*, eds Lewin G. R. Carter B. D. (Berlin: Springer), 497–512. 10.1007/978-3-642-45106-5_19 24668485

[B77] Schildt S. Endres T. Lessmann V. Edelmann E. (2013). Acute and chronic interference with BDNF/TrkB-signaling impair LTP selectively at mossy fiber synapses in the CA3 region of mouse hippocampus. *Neuropharmacology* 71 247–254. 10.1016/j.neuropharm.2013.03.041 23587649

[B78] Schlamp C. L. Montgomery A. D. Mac Nair C. E. Schuart C. Willmer D. J. Nickells R. W. (2013). Evaluation of the percentage of ganglion cells in the ganglion cell layer of the rodent retina. *Mol. Vis.* 19 1387–96. 23825918 PMC3695759

[B79] Sefton A. Dreher B. Harvey A. Martin P. (2015). “Visual system,” in *The Rat Nervous System*, ed. Paxinos G. (UK: Elsevier), 947–983.

[B80] Sefton A. Lam K. (1984). Quantitative and morphological studies on developing optic axons in normal and enucleated albino rats. *Exp. Brain Res.* 57 107–117. 6542867 10.1007/BF00231137

[B81] Shelton D. L. Sutherland J. Gripp J. Camerato T. Armanini M. P. Phillips H. S. (1995). Human trks: molecular cloning, tissue distribution, and expression of extracellular domain immunoadhesins. *J. Neurosci.* 15 477–491. 10.1523/jneurosci.15-01-00477.1995 7823156 PMC6578290

[B82] Simon D. K. O’Leary D. (1992). Development of topographic order in the mammalian retinocollicular projection. *J. Neurosci.* 12 1212–1232. 10.1523/jneurosci.12-04-01212.1992 1313491 PMC6575796

[B83] Spalding K. L. Rush R. A. Harvey A. R. (2004). Target-derived and locally derived neurotrophins support retinal ganglion cell survival in the neonatal rat retina. *Dev. Neurobiol.* 60 319–327. 10.1002/neu.20028 15281070

[B84] Spalding K. L. Tan M. M. Hendry I. A. Harvey A. R. (2002). Anterograde transport and trophic actions of BDNF and NT-4/5 in the developing rat visual system. *Mol. Cell. Neurosci.* 19 485–500. 10.1006/mcne.2001.1097 11988017

[B85] Tran N. M. Shekhar K. Whitney I. E. Jacobi A. Benhar I. Hong G. (2019). Single-cell profiles of retinal ganglion cells differing in resilience to injury reveal neuroprotective genes. *Neuron* 104 1039–1055.e12. 31784286 10.1016/j.neuron.2019.11.006 PMC6923571

[B86] Vaynman S. Ying Z. Gomez-Pinilla F. (2004). Hippocampal BDNF mediates the efficacy of exercise on synaptic plasticity and cognition. *Eur. J. Neurosci.* 20 2580–2590. 10.1111/j.1460-9568.2004.03720.x 15548201

[B87] Vecino E. Garcıìa-Grespo D. Garcıìa M. Martinez-Millán L. Sharma S. C. Carrascal E. (2002). Rat retinal ganglion cells co-express brain derived neurotrophic factor (BDNF) and its receptor TrkB. *Vision Res.* 42 151–157. 10.1016/s0042-6989(01)00251-6 11809469

[B88] Villegas-Pérez M. Vidal-Sanz M. Rasminsky M. Bray G. M. Aguayo A. J. (1993). Rapid and protracted phases of retinal ganglion cell loss follow axotomy in the optic nerve of adult rats. *J. Neurosci.* 24 23–36. 10.1002/neu.480240103 8419522

[B89] Voyatzis S. Muzerelle A. Gaspar P. Nicol X. (2012). Modeling activity and target-dependent developmental cell death of mouse retinal ganglion cells ex vivo. *PLoS One* 7:e31105. 10.1371/journal.pone.0031105 22363559 PMC3281910

[B90] Yang J. Siao C.-J. Nagappan G. Marinic T. Jing D. McGrath K. (2009). Neuronal release of proBDNF. *Nat. Neurosci.* 12 113–5. 10.1038/nn.2244 19136973 PMC2737352

[B91] Young R. W. (1984). Cell death during differentiation of the retina in the mouse. *J. Comp. Neurol.* 229 362–373. 10.1002/cne.902290307 6501608

[B92] Zhou X.-F. Rush R. A. (1996). Endogenous brain-derived neurotrophic factor is anterogradely transported in primary sensory neurons. *Neuroscience* 74 945–951. 10.1016/0306-4522(96)00237-0 8895863

